# A phase 1/2 study of azacitidine, venetoclax and pevonedistat in newly diagnosed secondary AML and in MDS or CMML after failure of hypomethylating agents

**DOI:** 10.1186/s13045-023-01476-8

**Published:** 2023-07-08

**Authors:** Nicholas J. Short, Muharrem Muftuoglu, Faustine Ong, Lewis Nasr, Walid Macaron, Guillermo Montalban-Bravo, Yesid Alvarado, Mahesh Basyal, Naval Daver, Courtney D. Dinardo, Gautam Borthakur, Nitin Jain, Maro Ohanian, Elias Jabbour, Ghayas C. Issa, Wei Qiao, Xuelin Huang, Rashmi Kanagal-Shamanna, Keyur P. Patel, Prithviraj Bose, Farhad Ravandi, Ricardo Delumpa, Regina Abramova, Guillermo Garcia-Manero, Michael Andreeff, Jorge Cortes, Hagop Kantarjian

**Affiliations:** 1 Department of Leukemia, Unit 428, The University of Texas MD Anderson Cancer Center, 1515 Holcombe Boulevard, Houston, TX 77030 USA; 2 Department of Biostatistics, The University of Texas MD Anderson Cancer Center, Houston, TX USA; 3 Department of Hematopathology, The University of Texas MD Anderson Cancer Center, Houston, TX USA; 4 Georgia Cancer Center, Augusta University, Augusta, GA USA

**Keywords:** Protein neddylation, Elderly, Myeloid diseases, Therapy, Clinical trial

## Abstract

**Background:**

Pevonedistat is a first-in-class, small molecular inhibitor of NEDD8-activating enzyme that has clinical activity in acute myeloid leukemia (AML) and myelodysplastic syndromes (MDS). Preclinical data suggest synergy of pevonedistat with azacitidine and venetoclax.

**Methods:**

This single-center, phase 1/2 study evaluated the combination of azacitidine, venetoclax and pevonedistat in older adults with newly diagnosed secondary AML or with MDS or chronic myelomonocytic leukemia (CMML) after failure of hypomethylating agents. Patients received azacitidine 75 mg/m^2^ IV on days 1–7, venetoclax at maximum dose of 200-400 mg orally on days 1–21 (AML cohort) or days 1–14 (MDS/CMML cohort) and pevonedistat 20 mg/m^2^ IV on days 1, 3 and 5 for up to 24 cycles. The primary endpoints for the phase 2 portion of the study were the CR/CRi rate in the AML cohort and the overall response rate (CR + mCR + PR + HI) in the MDS/CMML cohort.

**Findings:**

Forty patients were enrolled (32 with AML and 8 with MDS/CMML). In the AML cohort, the median age was 74 years (range 61–86 years), and 27 patients (84%) had at least one adverse risk cyto-molecular feature, including 15 (47%) with a *TP53* mutation or *MECOM* rearrangement; seventeen patients (53%) had received prior therapy for a preceding myeloid disorder. The CR/CRi rate was 66% (CR 50%; CRi 16%), and the median overall survival (OS) was 8.1 months. In the MDS/CMML cohort, 7 patients (87%) were high or very high risk by the IPSS-R. The overall response rate was 75% (CR 13%; mCR with or without HI 50%; HI 13%). The most common grade 3–4 adverse events were infection in 16 patients (35%), febrile neutropenia in 10 patients (25%) and hypophosphatemia in 9 patients (23%). In an exploratory analysis, early upregulation of NOXA expression was observed, with subsequent decrease in MCL-1 and FLIP, findings consistent with preclinical mechanistic studies of pevonedistat. Upregulation of CD36 was observed, which may have contributed to therapeutic resistance.

**Conclusions:**

The triplet combination of azacitidine, venetoclax and pevonedistat shows encouraging activity in this very poor-risk population of patients with AML, MDS or CMML.

*Trial registration* ClinicalTrials.gov (NCT03862157).

**Supplementary Information:**

The online version contains supplementary material available at 10.1186/s13045-023-01476-8.

## Introduction

Secondary acute myeloid leukemia (AML) is a heterogeneous subtype of AML that arises from an antecedent myeloid hematologic disorder or develops after exposure to chemotherapy or irradiation (also called “therapy-related AML”) [1, 2]. Compared to de novo AML, secondary AML is characterized by increased genomic complexity and enrichment of somatic mutations that are relatively resistant to chemotherapy, leading to poorer response to conventional therapies and shorter survival [2, 3]. Patients with secondary AML who have previously received hypomethylating agents (HMAs) or other forms of chemotherapy for an antecedent myeloid hematologic disorder (i.e., “treated secondary AML”) have a particularly poor prognosis [4, 5]. Similarly, patients with higher-risk myelodysplastic syndromes (MDS) that are resistant to or progress after HMA therapy also have poor outcomes, with a median overall survival (OS) of only 4–6 months, and there is no standard of care for this population [6, 7].

Older adults have an higher incidence of secondary AML and other adverse-risk genomic features that are associated with chemoresistance [2, 8, 9]. The combination of an HMA plus venetoclax is the current standard of care for older patients with newly diagnosed AML who are deemed unfit for intensive chemotherapy [10]. However, the outcomes with this approach are suboptimal in patients with adverse-risk cytomolecular features, where median overall survival (OS) is 6–8 months, with even worse outcomes in those with *TP53-*mutated AML or treated secondary AML [5, 10, 11]. While some retrospective and prospective studies have shown potential benefit of an HMA plus venetoclax in patients with MDS after HMA failure [12, 13], these findings have not yet been confirmed in randomized study and this approach is still considered investigational.

Pevonedistat is a first-in-class inhibitor of neural cell developmentally downregulated 8 (NEDD8)-activating enzyme (NAE) that catalyzes the rate-limiting step in the process of protein neddylation, a critical step in the degradation of a wide variety of cellular proteins that takes place upstream of the proteasome [14, 15]. Inhibition of NAE leads to diverse anti-leukemic cellular changes, including inhibition of nuclear factor-κB activity, induction of DNA damage, reactive oxygen species generation, downregulation of anti-apoptotic proteins and upregulation of pro-apoptotic proteins [15–17]. Preclinical studies suggest that pevonedistat synergizes with both azacitidine and venetoclax by upregulation of the pro-apoptotic protein NOXA; increased NOXA levels neutralize MCL-1, an anti-apoptotic protein that is the dominant resistance mechanism of venetoclax [18–20]. Some retrospective and prospective clinical studies have also suggested activity of the combination of azacitidine and pevonedistat in MDS, chronic myelomonocytic leukemia (CMML) and AML [21, 22].

Given the established clinical activity of azacitidine plus venetoclax as well as the preclinical and clinical data supporting the use of pevonedistat in myeloid malignancies, we designed a phase 1/2 study to evaluate the triplet combination of azacitidine, venetoclax and pevonedistat in patients with newly diagnosed secondary AML who are unfit for intensive chemotherapy and in patients with MDS or CMML after failure of hypomethylating agents.

## Methods

### Study design and participants

This was a single-center, phase 1/2 study to assess the efficacy and safety of the combination of azacitidine, venetoclax and pevonedistat in patients with newly diagnosed secondary AML or in MDS or CMML after failure of hypomethylating agents. This study was conducted at a single academic center (The University of Texas MD Anderson Cancer Center [UTMDACC]).

For the AML cohort, patients were required to have newly diagnosed AML with a history of MDS, MPN or MDS/MPN, MDS-related cytogenetics (other than del9q), morphological dysplasia in ≥ 50% cells in ≥ 2 myeloid lineage (unless accompanied by mutant *NPM1* or biallelic *CEBPA* mutations), and/or exposure to prior chemotherapy or radiation therapy for another malignancy; in this cohort, patients must also be considered unsuitable for intensive chemotherapy. Secondary AML subtypes for inclusion in this study were based on definitions of AML with myelodysplasia-related changes and therapy-related AML from the World Health Organization 2016 classification system of myeloid neoplasms [23]. For the MDS/CMML cohort, patients were required to have MDS or CMML with intermediate-1, intermediate-2 or high-risk disease by the International Prognostic Scoring System (IPSS) and have disease that did not respond, progressed or relapsed after at least 4 cycles of azacitidine and/or decitabine; in this cohort, patients with prior treatment with venetoclax or pevonedistat were ineligible. Additional eligibility criteria in both cohorts included: age ≥ 18 years, Eastern Cooperative Oncology Group (ECOG) performance status 0–2, total bilirubin ≤ 1.5 × upper limit of normal (ULN), alanine aminotransferase (ALT) and aspartate aminotransferase (AST) ≤ 2.5 × ULN, creatinine clearance ≥ 30 mL/minutes and white blood cell count < 50,000/µL. Key exclusion criteria included: extramedullary only disease, uncontrolled cardiopulmonary disease or hypertension, left ventricular ejection fraction < 50%, clinically significant prior or concurrent other malignancy, and use of strong CYP3A4 inducers within 14 days. Full inclusion and exclusion criteria are available in the protocol (see Additional file 1). This study was approved by the Institutional Review Board of UTMDACC. All patients provided informed consent according to institutional guidelines and the Declaration of Helsinki.

### Treatment regimen

In the AML cohort, cycle 1 consisted of azacitidine 75 mg/m^2^ intravenously (IV) or subcutaneously (SC) on days 1–7, venetoclax orally with ramp-up to a maximum dose of 200-400 mg (determined by the dose level in phase 1) on day 1–28 and pevonedistat 20 mg/m^2^ IV on days 1, 3 and 5. A bone marrow examination was performed on cycle 1, day 21 and venetoclax was held if bone marrow blasts < 5% or if aplastic. For cycle 2 and beyond, patients received azacitidine 75 mg/m^2^ IV or SC on days 1–7, venetoclax orally 200–400 mg on days 1–21, and pevonedistat 20 mg/m^2^ IV on days 1, 3 and 5. In the MDS/CMML cohort, all cycles consisted of azacitidine 75 mg/m^2^ IV or SC on days 1–7, venetoclax orally with ramp-up to 400 mg on day 1–14 and pevonedistat 20 mg/m^2^ IV on days 1, 3 and 5. The venetoclax dose was reduced by 50% for patients receiving a moderate CYP3A4 inhibitor and by 75% for patients receiving a strong CYP3A4 inhibitor (with the exception of posaconazole for which venetoclax was reduced by 83%, e.g., from 400 to 70 mg daily). In both cohorts, the regimen consisted of up to 24 cycles of azacitidine, venetoclax and pevonedistat. Each cycle was anticipated to be 28 days in length, although cycle delays were allowed due to delayed count recovery or intercurrent illness.

### Outcomes

The primary endpoint of the phase 1 portion of the study (AML only) was the dose-limiting toxicity (DLT) of the combination regimen. The primary endpoint for the phase 2 portion of the AML cohort was the complete remission (CR) or CR with incomplete hematological recovery (CRi) rate, and the primary endpoint for the phase 2 portion of the MDS/CMML cohort was the composite rate of CR, marrow CR (mCR), partial remission (PR) and hematologic improvement (HI). Secondary endpoints included the CR rate, composite rate of CR, CRi, PR, and morphologic leukemia-free state (MLFS) (AML cohort only), measurable residual disease (MRD) negativity rate by flow cytometry (AML cohort only), time to AML transformation (MDS/CMML cohort only), relapse-free survival (RFS), overall survival (OS) and safety of the regimen.

AML responses were defined according to the European LeukemiaNet 2017 guidelines [24]. MDS responses were defined according MDS or MDS/MPN International Working Group recommendations [25, 26]. RFS was calculated from the time of response until relapse or death from any cause, censored if alive at last follow-up. OS was calculated from the time of treatment initiation until death from any cause, censored if alive at last follow-up. Safety was assessed with the Common Terminology Criteria for Adverse Events (CTCAE) version 4.03.

MRD assessment was performed on fresh bone marrow aspiration samples using 8-color multiparameter flow cytometry as described previously [27]. The sensitivity of this assay is 0.1% or better. Mutation analysis was performed on bone marrow specimens 81-gene targeted next-generation sequencing panel as previously described [28, 29].

### Exploratory biomarker analysis

Serial peripheral blood and bone marrow samples were collected from patients in the AML cohort for cytometry by time of flight (CyTOF) analysis using a 51-parameter, leukemia-focused panel. A comprehensive CyTOF analysis of sequentially collected samples was performed, as previously described [30, 31], with an aim to interrogate changes in signaling pathways over the course of therapy. Details are in the Additional file 1: Methods.

### Statistical analysis

The AML arm consisted of both phase 1 and phase 2 portions. The phase 1 portion was conducted using a standard “3 + 3” design and evaluated venetoclax at a dose a maximum dose of 200 mg (dose level 0) and 400 mg (dose level + 1). After completion of phase 1, up to 22 additional patients were to be enrolled in the phase 2 portion of the study (for a total of evaluable 28 patients treated at the recommended phase 2 dose). Interim monitoring rules for efficacy and toxicity were used throughout the phase 2 portion. The study was continuously monitored for efficacy and treatment-related toxicities using a Bayesian design [32, 33]. The regimen was considered promising if the CR rate was at ≥ 40% and the grade ≥ 3 non-hematologic toxicity rate was < 20% within 6 cycles of treatment. A CR rate of 40% was targeted to reflect a CR rate that was similar to or better than the 36.7% CR rate reported in the VIALE-A study, acknowledging that the patients in our study were expected to generally have poorer-risk disease features than those enrolled in VIALE-A [10]. Patients in both the phase 1 and 2 portions of the study were included in the primary efficacy analyses.

The MDS/CMML arm was opened after safety of the triplet regimen had been established in AML, and it consisted of a phase 2 portion only. Continuous monitoring for efficacy and treatment-related toxicities was performed as described above [32, 33]. The regimen was considered promising if the overall response rate (CR + mCR + PR + HI) was ≥ 25% and the grade ≥ 3 non-hematologic toxicity rate was < 20% within 6 cycles of treatment. Initially, 20 patients were planned to be enrolled in the MDS/CMML post-HMA failure cohort. However, due to lack of benefit of pevonedistat in a randomized phase 3 study of azacitidine versus azacitidine plus pevonedistat in higher-risk MDS/CMML or low-blast AML [34], the MDS/CMML cohort was terminated after 8 patients had been enrolled.

Patient characteristics were summarized using the median (range) for continuous variables and the frequencies (percentages) for categorical variables. Remission duration, RFS and OS were calculated with Kaplan–Meier estimates, and survival estimates were compared with the log-rank test. The data cutoff for this analysis was December 1, 2022. The data analyses were carried out using GraphPad Prism 9. This study was registered at ClinicalTrials.gov (NCT03862157).

## Results

### Patient characteristics of the AML cohort

**Fig. 1:**
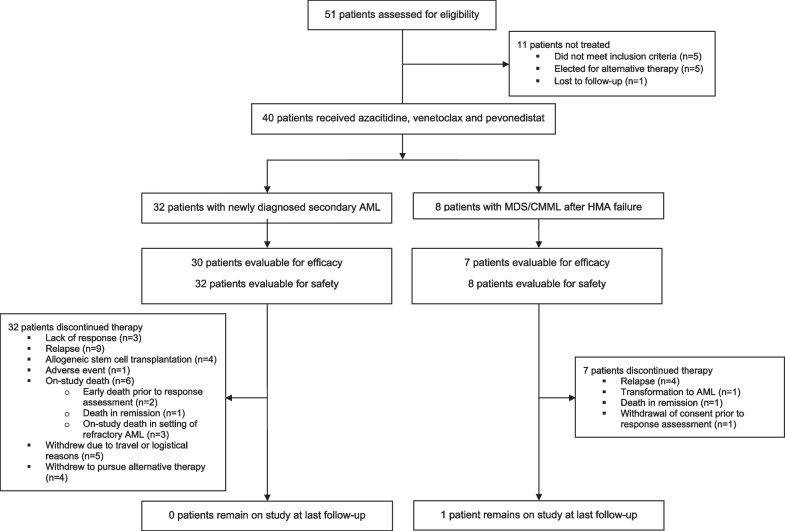
CONSORT diagram for the study population

Between March 2019 and September 2021, 32 patients with newly diagnosed secondary or therapy-related AML were treated (Table 1; Fig. 1). The median age was 74 years (range 61–86 years), and 14 patients (44%) were ≥ 75 years of age. Six patients (19%) had therapy-related AML without a history of MDS, MDS/MPN or MPN, 22 patients (69%) had a prior diagnosis of myeloid disorder (4 of whom were also therapy-related), and 4 patients (13%) had MDS-related cytogenetics but no clinical history of preceding myeloid disorder. Overall, 17 patients (53%) had treated secondary AML (i.e., prior HMA and/or chemotherapy for a preceding myeloid disorder). Cytogenetics were adverse in 21 patients (66%), including 12 (38%) patients with complex karyotype and 4 (13%) with *MECOM* inversion or translocation, 1 of whom also had a complex karyotype. Twenty-seven patients (84%) had adverse risk cytomolecular features by ELN 2017. The most common identified mutations were *TP53* and *TET2* in 11 patients each (34%), *RUNX1* in 10 patients (31%) and *KRAS* and/or *NRAS* in 8 patients (25%).

**Table 1:** Baseline characteristics of the AML cohort (N = 32)

Characteristic	Value
Age (years)	74 [61–86]
≥ *75 years*	14 (44)
WBC (× 10^9^/L)	2.5 [0.9–26.9]
Platelets (× 10^9^/L)	30.0 [8.0–354.0]
Bone marrow blasts (%)	29.5 [14.0–86.0]
AML subtype	
*Therapy-related AML (without antecedent myeloid disorder)*	6 (19)
*Prior MDS, MDS/MPN, or MPN*	22 (69)
*AML-MRC (without antecedent myeloid disorder)*	4 (13)
Prior HMA or chemotherapy exposure for antecedent myeloid disorder	17 (53)
Cytogenetics	
*Complex*	12 (38)
MECOM-rearranged*	4 (13)
*Other adverse (non-complex, non-MECOM)*	6 (19)
*Diploid*	6 (19)
*Others (non-adverse)*	4 (13)
Mutations^#^	
*ASXL1*	7 (22)
*BCOR*	4 (13)
*CEBPA*	7 (22)
*CSF3R*	3 (10)
*DNMT3A*	5 (16)
*EZH2*	3 (10)
*KRAS/NRAS*	8 (25)
*NF1*	6 (19)
*PTPN11*	3 (10)
*RUNX1*	10 (31)
*SRSF2*	5 (16)
*STAG2*	4 (13)
*TET2*	11 (34)
*TP53*	11 (34)
*ZRSR2*	3 (10)
ELN 2017 cytomolecular risk	
*Favorable*	0
*Intermediate*	5 (16)
*Adverse*	27 (84)

Continuous variables are listed as median [range] and categorical variables as n (%) or n/N (%)

WBC, white blood cells; HMA, hypomethylating agent; MDS, myelodysplastic syndrome; MPN, myeloproliferative neoplasm; MRC, myelodysplasia-related changes; ELN, European LeukemiaNet

^*^One patient had *MECOM* rearrangement with complex cytogenetics but is included only in the *MECOM*-rearranged group in the table

^#^Mutations detected in at least 3 patients are shown in the table

### Phase 1 results

Ten patients with newly diagnosed secondary or therapy-related AML were enrolled into the phase 1 portion of the study (3 at dose level 0 with venetoclax 200 mg and 7 at dose level 1 with venetoclax 400 mg). One patient who received the 400 mg dose of venetoclax died on cycle 1, day 14 from pneumonia in the setting of underlying chronic pulmonary fibrosis; this death was considered unrelated to the study drugs so was not considered to be a DLT. Overall, no DLTs were observed at the 2 venetoclax doses evaluated, and a daily dose of 400 mg of venetoclax was chosen as the recommended phase 2 dose for further study.

### Response and survival outcomes in the AML cohort

Sixteen patients (50%) achieved CR and 5 patients (16%) achieved CRi, for a composite CR/CRi rate of 66%. An additional 3 patients (9%) achieved MLFS, and 1 patient (3%) achieved PR as best response. There were 2 early deaths, one on cycle 1, day 14 from pneumonia and one on cycle 1, day 26 from sepsis. Responses by subgroups are shown in Table 2. The CR/CRi rates in patients with treated secondary AML and those without prior HMA or chemotherapy exposure were 59% (10/17) and 73% (11/15), respectively. CR/CRi rates for patients with poor-risk and non-poor-risk cytogenetics were 52% (11/21) and 91% (10/11), respectively. Among the 21 patients who achieved CR/CRi as best response, 10 (48%) achieved MRD negativity by multiparameter flow cytometry.

**Table 2:** CR/CRi rates by subgroup

Subgroup	n/N (%)
AML subtype	
*Therapy-related AML*^*#*^	7/10 (70)
*AML arising from prior MDS, MDS/MPN, or MPN (without prior therapy)*	4/5 (80)
*AML arising from prior MDS, MDS/MPN, or MPN (with prior HMA or chemotherapy)*	10/17 (59)
AML-MRC^*^	3/4 (75)
Cytogenetics	
*Poor-risk cytogenetics*	11/21 (52)
*Non-poor-risk cytogenetics*	10/11 (91)
ELN 2017 cytomolecular risk	
*Intermediate*	5/5 (100)
*Adverse*	16/27 (59)
*TP53-mutated*	7/11 (64)
*MECOM-rearranged*	2/4 (50)

MDS, myelodysplastic syndrome; MPN, myeloproliferative neoplasm; MRC, myelodysplasia-related changes; HMA, hypomethylating agent; ELN, European LeukemiaNet

^#^Includes 4 patients with AML arising from preceding therapy-related myeloid disorder

^*^Excluding patients with therapy-related AML or those with preceding myeloid disorder

**Fig. 2:**
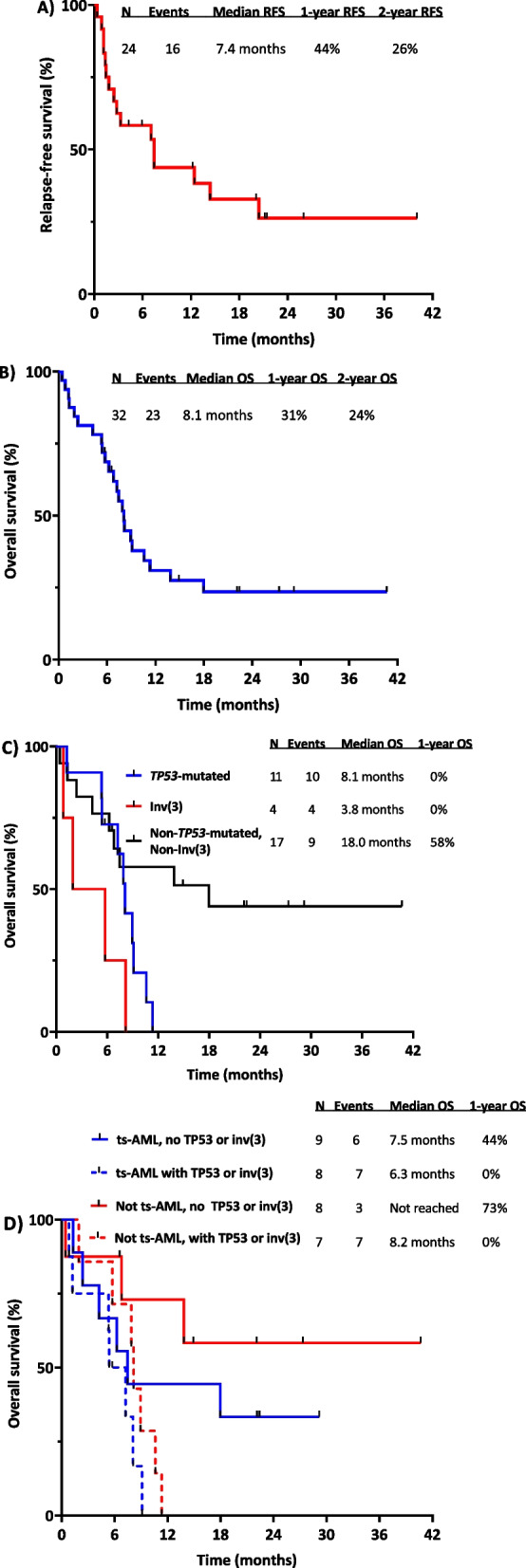
Outcomes of the AML cohort. **A** Relapse-free survival for the entire cohort, **B** overall survival for the entire cohort, **C** overall survival by presence of *TP53* mutation or *MECOM* rearrangement, and **D** overall survival by clinical history of prior hypomethylating agent or chemotherapy exposure for antecedent hematologic malignancy and by presence of *TP53* mutation or *MECOM* rearrangement

The median number of cycles received in the AML cohort is 2.5 (range 1–13 cycles). The median follow-up is 22.4 months (range 0.4–40.7 months). Five patients (16% overall and 24% of patients achieving CR/CRi) proceeded to allogeneic SCT in first remission. Three of the transplanted patients were still alive at last follow-up; 1 patient with *MECOM-*rearranged AML who achieved MRD-positive CRi as best response relapsed 3.8 months post-SCT and 1 patient with *TP53-*mutated treated secondary AML who achieved MRD-positive CRi as best response relapsed 3.1 months post-SCT, both of whom subsequently died from leukemia-related complications. As of the data cutoff, 13 (41%) patients have relapsed, and 23 (72%) patients have died. There are no patients in the AML cohort still receiving protocol therapy as of the data cutoff. Among responders, the median duration of response was 7.4 months. The median RFS was 7.4 months, and the estimated 2-year RFS was 26% (Fig. 2A). The median OS was 8.1 months, and the estimated 2-year OS was 24% (Fig. 2B). In a post hoc analysis, the outcomes of patients with adverse risk karyotype were significantly inferior to those without an adverse risk karyotype (median OS 7.2 months versus not reached and 2-year OS 10% versus 51%, respectively; *P* = 0.02) (Additional file 1 Figure s1). The worst outcomes were seen in patients with either *TP53*-mutated AML (n = 11) or *MECOM-*rearranged AML (n = 4). In these subgroups, the median OS was 8.1 months and 3.8 months, respectively, and no patient in either group was alive beyond 1 year. In contrast, the median OS for patients without a *TP53* mutation or *MECOM* rearrangement was 18.0 months, and the 2-year OS was 44% (Fig. 2C). The presence of a *TP53* mutation or *MECOM* rearrangement appeared to have a more substantial impact on survival that did history of prior treatment of an antecedent myeloid disorder (Fig. 2D).

### Patient characteristics of the MDS/CMML cohort

Between January 2021 and August 2021, 8 patients with MDS/CMML after failure of HMAs were treated. The baseline characteristics and responses of the MDS cohort are shown in Table 3. The median age was 73 years (range 49–83 years). Six patients had MDS and 2 patients had CMML. The median bone marrow blast percentage was 7.5% (range 2%-15%). Five patients had received 1 prior therapy, 2 had received 2 prior therapies, and 1 had received 3 prior therapies. One patient had undergone prior allogeneic SCT. Half of patients had adverse cytogenetics, and 7/8 (87%) were high or very high risk by IPSS-R.

**Table 3:** Baseline characteristics and responses of the MDS cohort (N = 8)

Patient	Age (years)	Disease subtype	Number of prior lines of therapy	Baseline bone marrow blasts	IPSS-R risk	Mutations	Best response	Duration of response (months)
#1	59	MDS	1	15%	Very high	*TP53, BRINP3*	Not evaluable	N/A
#2	71	MDS	2	4%	High	*ASXL1, NF1, RUNX1, SF3B1, PHF6*	Stable disease	5.1
#3	83	CMML	1	6%	Intermediate	*SF3B1, SRSF2, ASXL1, RUNX1, SETBP1, PRPF40B*	mCR	4.4
#4	74	MDS	1	3%	Very high	*TP53*	CR	14.8 (ongoing)
#5	49	CMML	2	11%	Very high	*ASXL1, PTPN11, U2AF1*	mCR	2.8
#6	72	MDS	1	2%	High	*EZH2, SF3B1, TET2, RAD21*	HI-P	10.9
#7	76	MDS	1	9%	High	*TP53, ASXL2, ETV6, SETBP1, ZRSR2*	mCR + HI-E + HI-N	8.0
#8	78	MDS	3	14%	Very high	*IDH2, SRSF2, FLT3-*D835*, RUNX1*	mCR + HI-N	3.9

Abbreviations: MDS, myelodysplastic syndrome; CMML, chronic myelomonocytic leukemia; IPSS-R, Revised International Prognostic Scoring System; CR, complete response; mCR, marrow complete response; HI, hematologic improvement; HI-E, HI with erythroid response; HI-N, HI with neutrophil response; HI-P, HI with platelet response

### Response and survival outcomes in the MDS/CMML cohort

Among 8 patients with MDS/CMML, 1 patient (13%) achieved CR, 4 patients (50%) achieved mCR (2 of whom also achieved HI), and 1 patient (13%) achieved HI, for an overall response rate of 75%. One patient with low blast percentage at start (4%) received 6 cycles and achieved stable disease at best response. One patient withdrew consent on day 5 and opted for supportive care and therefore was unevaluable for response.

The median number of cycles received in the MDS/CMML cohort is 5 (range 1–16 cycles). The median follow-up is 17.6 months (range 1.0–19.3 months). No patients proceeded to subsequent allogeneic SCT. As of the data cutoff, 4 of the 5 responding patients have relapsed and 6 patients have died. One patient with complex cytogenetics with a *TP53* mutation is still receiving protocol therapy as of the data cutoff; this patient achieved CR has ongoing response of 14.8 months. The median OS was 10.9 months, and the estimated 1-year OS was 38% (Additional file 1: Figure s2).

### Safety

Non-hematologic adverse events across both cohorts are shown in Table 4. Six patients (15%) experienced grade 1–2 hypophosphatemia, and 9 patients (23%) experienced grade 3 hypophosphatemia; in all cases, this was considered possibly related to pevonedistat. Hypophosphatemia was transient and manageable with oral and/or IV phosphorus repletion. Three patients (8%) experienced grade 3 AST/ALT elevation and 1 patient (2%) experienced grade 4 AST/ALT elevation, both of which were considered possibly related to pevonedistat. Three patients (8%) experience grade 3 hyperbilirubinemia (in 1 case considered possibly related to pevonedistat). One patient developed grade 4 transaminase elevation, grade 4 acute kidney injury and grade 3 hyperbilirubinemia following the first dose of the study drugs, all of which were considered possibly related to pevonedistat. This patient recovered with supportive measures but was taken off study due to concern for pevonedistat-induced toxicity. This patient subsequently received azacitidine × 5 days and achieved CR with MRD negativity by flow cytometry, despite the minimal therapy received.

**Table 4:** Non-hematologic adverse events observed in either cohort (AML and MDS/CMML), regardless of causality

Parameter	Grade 1–2	Grade 3	Grade 4	Grade 5
Acute coronary syndrome	0	0	1 (2%)	0
Acute kidney injury	0	1 (2%)	1 (2%)	0
Anorexia	2 (5%)	1 (2%)	0	0
Atrial fibrillation	0	1 (2%)	0	0
Cholecystitis	0	2 (5%)	0	0
Dehydration	0	1 (2%)	0	0
Delirium	0	1 (2%)	0	0
Disease progression	0	0	0	1 (2%)
Enterocolitis	0	3 (8%)	0	0
Fatigue	4 (10%)	0	0	0
Febrile neutropenia	0	9 (23%)	1 (2%)	0
Fluid overload	0	1 (2%)	0	0
Generalized muscle weakness	0	1 (2%)	0	0
Gastrointestinal hemorrhage	0	0	0	1 (2%)
Hyperglycemia	0	3 (8%)	0	0
Hypokalemia	0	2 (5%)	0	0
Hypophosphatemia	6 (15%)	9 (23%)	0	0
Hyponatremia	0	1 (2%)	0	0
Hypotension	1 (2%)	1 (2%)	0	0
Increased ALT/AST	4 (10%)	3 (8%)	1 (2%)	0
Increased bilirubin	2 (5%)	3 (8%)	0	0
Infection	0	16 (35%)	0	1 (2%)
Insomnia	0	1 (2%)	0	0
Intracranial hemorrhage	0	0	1 (2%)	0
Multi-organ failure	0	0	0	1 (2%)
Nausea/vomiting	4 (10%)	2 (6%)	0	0
Oral mucositis	1 (2%)	1 (2%)	0	0
Pain (extremities/back)	0	3 (8%)	0	0
Pneumonitis	0	2 (5%)	0	0
Rash (acneiform)	0	1 (2%)	0	0
Sepsis	0	0	2 (5%)	0
Small bowel obstruction	0	2 (5%)	0	0
Stroke	0	0	0	1 (2%)
Sudden death	0	0	0	2 (5%)
Syncope	0	1 (2%)	0	0

ALT, alanine aminotransferase; AST, aspartate aminotransferase

Data are n (%). Any grade 1–2 adverse event occurring in ≥ 10% of patients, and all grade 3, 4 and 5 adverse events are included

Across both cohorts, 11 patients (28%) had at least one dose reduction or interruption of one or more study drugs, 9 patients (28%) in the AML cohort and 2 patients (25%) in the MDS/CMML cohort. Overall, 5 patients (13%) had a dose reduction of azacitidine, 6 patients (15%) had a dose reduction of venetoclax, and no patients had a dose reduction of pevonedistat. The most common reason for dose reduction of azacitidine or venetoclax was myelosuppression (accounting for 82% of dose reductions). One patient (3%) had a dose interruption of azacitidine, no patients had a dose interruption of venetoclax, and 5 patients (13%) had a dose interruption of pevonedistat. The most common reasons for dose interruptions of pevonedistat were transient hypophosphatemia (in 3 cases) and elevated transaminases (in 2 cases). One patient discontinued therapy due to treatment-related toxicity (transaminase elevation, hyperbilirubinemia and acute kidney injury, possibly related to pevonedistat).

There were 7 on-study deaths (6 in the AML cohort and 1 in the MDS/CMML cohort). In the AML cohort, the 30-day and 60-day mortality rates were 6% and 16%, respectively. In the MDS/CMML cohort, the 30-day and 60-day mortality rates were 0% and 13%, respectively.

### CyTOF analysis of pretreatment and posttreatment samples

CyTOF was performed on patients in the AML cohort using paired pretreatment and cycle 1, day 2 samples (n = 11) and using pretreatment and cycle 1, day 21 samples (n = 16). We utilized UMAP analysis to dissect the leukemia proteomic landscape and identify AML blasts and healthy non-malignant cells in the leukemia compartment (Additional file 1: Figure s3A). We then performed differential expression analysis to identify key features that were differentially regulated with treatment in AML blasts and in the monocytic population. On day 2 of therapy, NOXA expression was significantly increased in both the blast and monocytic populations, CD36 was increased in blasts, and pNRF2 was reduced in the monocytic population (*P* < 0.005 for all) (Additional file 1: Figure s3B). On day 21 of therapy, MCL-1, FLIP and c-MYC were significantly decreased and CD36 was significantly increased in the blast population (*P* < 0.005 for all) (Additional file 1: Figure s4). On day 21, MCL-1 and pNRF2 were significantly reduced in the monocytic population (*P* < 0.005 for all). We did not observe any significant compensatory changes in either BCL-2 or BCL-xL expression in either AML blasts or monocytic cells at either time point (data now shown).

## Discussion

Azacitidine plus venetoclax is standard of care for older adults with newly diagnosed AML who are unfit for intensive chemotherapy, although the outcomes for the substantial proportion of these patients who whose AML has adverse cytomolecular features remain suboptimal [10]. This study of azacitidine, venetoclax and pevonedistat was designed for patients with newly diagnosed secondary AML, a subtype of AML that is enriched with poor-risk cytomolecular features. In this poor-risk population, azacitidine, venetoclax and pevonedistat resulted in a CR/CRi rate of 66%, although responses were relatively short-lived, with a median duration of response of 7.4 months and median OS of 8.2 months. While these response duration and survival outcomes are numerically inferior to those reported in the global population of the VIALE-A study, they are largely consistent with expectations with azacitidine and venetoclax in patients with poor-risk cytogenetics or molecular mutations. [10]

Overall 84% of patients in the AML cohort had one or more poor-risk cytogenetic or molecular feature, including 34% with a *TP53* mutation and 13% with a *MECOM* rearrangement, two of the genomic features that are associated with the worst outcomes in AML [35, 36]. Within this population of patients with secondary AML, our studies confirm the strong adverse prognostic impact of these alterations. Among the 15 patients who had either a TP53 mutation or *MECOM* rearrangement, the 1-year OS was 0%; in contrast, patients without either of those abnormalities had a median OS of 18.0 months and a 2-year OS of 44%. Lack of meaningful duration of response in patients with *TP53-*mutated or *MECOM*-rearranged AML suggests that the addition of pevonedistat was unable to overcome the adverse prognostic impact of these alterations. The lack of clinical benefit of pevonedistat in *TP53*-mutated AML was also recently reported [37]. Conversely, the clinical activity and durations of response with this triplet regimen in patients without a *TP53* mutation or *MECOM* rearrangement were encouraging.

This study was also enriched with patients with treated secondary AML (accounting for 53% of the AML cohort). In one retrospective analysis, only 24% of older adults with treated secondary AML achieved CR/CRi with frontline induction therapy, which translated to a dismal median OS of 4.7 months [4]. The outcomes of these patients were akin to those with other high-risk features, including monosomy 5 and/or 7, *TP53* mutations or *MECOM* rearrangements. While the optimal treatment for these patients remains uncertain, lower-intensity therapy plus venetoclax is likely superior to intensive chemotherapy at the time of AML transformation, particularly for those without an adverse-risk karyotype [5]. In our study, the CR/CRi rate in patients with treated secondary AML was 59%, which compares favorably to historical expectations, although OS was still modest, highlighting the poor outcomes of this subtype of AML.

Effective treatment options for patients with MDS or CMML after HMA failure are limited. While previous studies have suggested clinical activity of HMA plus venetoclax, there is still no standard of care in this population [12, 13]. Among the 8 patients treated in the MDS/CMML arm of this study, the overall response rate was 75%, and the median OS was 10.9 months. While the initial activity of this regimen appeared promising in this setting, the study was stopped early due to negative results from the PANTHER study, a randomized phase 3 study comparing azacitidine plus pevonedistat to azacitidine alone in patients with newly diagnosed higher-risk MDS, CMML or AML with 20–30% blasts [34]. Despite initial promising results from a smaller, randomized phase 2 study in this same population [22], the phase 3 study failed to show a benefit of pevonedistat in the frontline treatment of these diseases. While it remains possible that pevonedistat could provide additive benefit in a triplet combination in MDS/CMML due to its established preclinical synergy with venetoclax, the small numbers accrued on this study prevent any definitive conclusions about its relative contribution to the responses observed.

Using CyTOF analysis, we observed an early increase in NOXA levels, followed decrease in MCL-1 levels, a finding that is consistent with preclinical data suggesting that pevonedistat may synergize with azacitidine and venetoclax by increasing NOXA levels, leading to downstream neutralization of MCL-1 [20]. FLIP, another anti-apoptotic protein, was also decreased in the blast compartment on day 21, consistent with preclinical data suggesting that inhibition of protein neddylation should decrease stability of FLIP. Studies comparing these findings to HMA plus venetoclax, without pevonedistat, are needed to determine the potential contribution of pevonedistat to these observed changes in protein expression. Interestingly, we also observed a sharp increase in levels of CD36 at the end of the first cycle of treatment. Fatty acid metabolism and CD36 expression, a fatty acid transporter, have been reported to mediate resistance to chemotherapy and/or venetoclax in AML [38, 39]. Future studies should further clarify the potential role of CD36 in mediating resistance to HMA plus venetoclax-based regimens.

Across both cohorts, the addition of pevonedistat was safe and did not appear to impair delivery of the intended doses of azacitidine or venetoclax. Hypophosphatemia, a known pevonedistat-induced electrolyte abnormality, was observed in 47% of patients but was manageable with oral or IV supplementation. Myelosuppression with the triplet regimen appeared consistent with historical expectations with azacitidine plus venetoclax. The lack of myelosuppression with pevonedistat is supported by two randomized studies in MDS/CMML of azacitidine plus pevonedistat versus azacitidine alone, where the pevonedistat-containing arm had a similar incidence of hematologic toxicity and was able to maintain dose intensity of azacitidine [22, 34]. Thus, the toxicity profile of pevonedistat makes it a potentially attractive non-myelosuppressive agent for combination studies in hematologic malignancies. It should be noted, however, that longitudinal quality-of-life assessments were not performed in our study, which is a limitation.

In summary, the triplet combination of azacitidine, venetoclax and pevonedistat was active in this very poor-risk population of older adults with newly diagnosed secondary AML and in patients with MDS or CMML after HMA failure. While the response rates and survival in patients with MDS or CMML after HMA failure are encouraging, the added clinical benefit of pevonedistat in this setting is questionable considering a negative randomized study of pevonedistat in these diseases. A randomized phase 2 study of azacitidine, venetoclax and pevonedistat versus azacitidine and venetoclax in patients with newly diagnosed AML who are unfit for intensive chemotherapy has fully accrued (NCT04266795) and will help to clarify the potential role of pevonedistat in this population.

## Supplementary Information

**Additional file 1. **Supplemental methods and figures.

## Data Availability

The datasets used and/or analyzed during the current study are available from the corresponding author upon reasonable request.

## Abbreviations

ALT: Alanine aminotransferase
AML: Acute myeloid leukemia
AST: Aspartate aminotransferase
CMML: Chronic myelomonocytic leukemia
CR: Complete remission
CRi: Complete remission with incomplete hematological recovery
CTCAE: Common Terminology Criteria for Adverse Events
CyTOF: Cytometry by time of flight
DLT: Dose-limiting toxicity
ECOG: Eastern Cooperative Oncology Group
HI: Hematologic improvement
HMA: Hypomethylating agent
IPSS: International Prognostic Scoring System
IV: Intravenous
mCR: Marrow complete remission
MDS: Myelodysplastic syndrome
MLFS: Morphologic leukemia-free state
MRD: Measurable residual disease
NAE: Nedd8-activating enzyme
OS: Overall survival
PR: Partial remission
RFS: Relapse-free survival
SC: Subcutaneous
UTMDACC: The University of Texas MD Anderson Cancer Center
ULN: Upper limit of normal

## References

[1] Stone RM, Mazzola E, Neuberg D (2015). Phase III open-label randomized study of cytarabine in combination with amonafide L-malate or daunorubicin as induction therapy for patients with secondary acute myeloid leukemia. J Clin Oncol.

[2] Lindsley RC, Mar BG, Mazzola E (2015). Acute myeloid leukemia ontogeny is defined by distinct somatic mutations. Blood.

[3] Granfeldt Ostgard LS, Medeiros BC, Sengelov H (2015). Epidemiology and clinical significance of secondary and therapy-related acute myeloid leukemia: a national population-based cohort study. J Clin Oncol.

[4] Boddu P, Kantarjian HM, Garcia-Manero G (2017). Treated secondary acute myeloid leukemia: a distinct high-risk subset of AML with adverse prognosis. Blood Adv.

[5] Short NJ, Venugopal S, Qiao W (2022). Impact of frontline treatment approach on outcomes in patients with secondary AML with prior hypomethylating agent exposure. J Hematol Oncol.

[6] Jabbour E, Garcia-Manero G, Batty N (2010). Outcome of patients with myelodysplastic syndrome after failure of decitabine therapy. Cancer.

[7] Prebet T, Gore SD, Esterni B (2011). Outcome of high-risk myelodysplastic syndrome after azacitidine treatment failure. J Clin Oncol.

[8] Podoltsev NA, Stahl M, Zeidan AM, Gore SD (2017). Selecting initial treatment of acute myeloid leukaemia in older adults. Blood Rev.

[9] Creutzig U, Zimmermann M, Reinhardt D (2016). Changes in cytogenetics and molecular genetics in acute myeloid leukemia from childhood to adult age groups. Cancer.

[10] DiNardo CD, Jonas BA, Pullarkat V (2020). Azacitidine and venetoclax in previously untreated acute myeloid leukemia. N Engl J Med.

[11] Kim K, Maiti A, Loghavi S (2021). Outcomes of TP53-mutant acute myeloid leukemia with decitabine and venetoclax. Cancer.

[12] Ball BJ, Famulare CA, Stein EM (2020). Venetoclax and hypomethylating agents (HMAs) induce high response rates in MDS, including patients after HMA therapy failure. Blood Adv.

[13] Zeidan AM, Borate U, Pollyea DA (2023). A phase 1b study of venetoclax and azacitidine combination in patients with relapsed or refractory myelodysplastic syndromes. Am J Hematol.

[14] Soucy TA, Smith PG, Rolfe M (2009). Targeting NEDD8-activated cullin-RING ligases for the treatment of cancer. Clin Cancer Res.

[15] Soucy TA, Smith PG, Milhollen MA (2009). An inhibitor of NEDD8-activating enzyme as a new approach to treat cancer. Nature.

[16] Swords RT, Erba HP, DeAngelo DJ (2015). Pevonedistat (MLN4924), a First-in-Class NEDD8-activating enzyme inhibitor, in patients with acute myeloid leukaemia and myelodysplastic syndromes: a phase 1 study. Br J Haematol.

[17] Swords RT, Kelly KR, Smith PG (2010). Inhibition of NEDD8-activating enzyme: a novel approach for the treatment of acute myeloid leukemia. Blood.

[18] Bose P, Gandhi V, Konopleva M (2017). Pathways and mechanisms of venetoclax resistance. Leuk Lymphoma.

[19] Cojocari D, Smith BN, Purkal JJ (2022). Pevonedistat and azacitidine upregulate NOXA (PMAIP1) to increase sensitivity to venetoclax in preclinical models of acute myeloid leukemia. Haematologica.

[20] Knorr KL, Schneider PA, Meng XW (2015). MLN4924 induces Noxa upregulation in acute myelogenous leukemia and synergizes with Bcl-2 inhibitors. Cell Death Differ.

[21] Swords RT, Coutre S, Maris MB (2018). Pevonedistat, a first-in-class NEDD8-activating enzyme inhibitor, combined with azacitidine in patients with AML. Blood.

[22] Sekeres MA, Watts J, Radinoff A (2021). Randomized phase 2 trial of pevonedistat plus azacitidine versus azacitidine for higher-risk MDS/CMML or low-blast AML. Leukemia.

[23] Arber DA, Orazi A, Hasserjian R (2016). The 2016 revision to the World Health Organization classification of myeloid neoplasms and acute leukemia. Blood.

[24] Dohner H, Estey E, Grimwade D (2017). Diagnosis and management of AML in adults: 2017 ELN recommendations from an international expert panel. Blood.

[25] Cheson BD, Greenberg PL, Bennett JM (2006). Clinical application and proposal for modification of the International Working Group (IWG) response criteria in myelodysplasia. Blood.

[26] Savona MR, Malcovati L, Komrokji R (2015). An international consortium proposal of uniform response criteria for myelodysplastic/myeloproliferative neoplasms (MDS/MPN) in adults. Blood.

[27] Ravandi F, Jorgensen J, Borthakur G (2017). Persistence of minimal residual disease assessed by multiparameter flow cytometry is highly prognostic in younger patients with acute myeloid leukemia. Cancer.

[28] Short NJ, Kantarjian HM, Loghavi S (2019). Treatment with a 5-day versus a 10-day schedule of decitabine in older patients with newly diagnosed acute myeloid leukaemia: a randomised phase 2 trial. Lancet Haematol.

[29] Luthra R, Patel KP, Reddy NG (2014). Next-generation sequencing-based multigene mutational screening for acute myeloid leukemia using MiSeq: applicability for diagnostics and disease monitoring. Haematologica.

[30] Muftuoglu M, Olson A, Marin D (2018). Allogeneic BK virus-specific T cells for progressive multifocal leukoencephalopathy. N Engl J Med.

[31] Muftuoglu M, Li L, Liang S (2021). Extended live-cell barcoding approach for multiplexed mass cytometry. Sci Rep.

[32] Thall PF, Wooten LH, Tannir NM (2005). Monitoring event times in early phase clinical trials: some practical issues. Clin Trials.

[33] Thall PF, Simon RM, Estey EH (1995). Bayesian sequential monitoring designs for single-arm clinical trials with multiple outcomes. Stat Med.

[34] Adès L, Girshova L, Doronin VA (2022). Pevonedistat plus azacitidine vs azacitidine alone in higher-risk MDS/chronic myelomonocytic leukemia or low-blast-percentage AML. Blood Adv.

[35] Kadia TM, Jain P, Ravandi F (2016). TP53 mutations in newly diagnosed acute myeloid leukemia: Clinicomolecular characteristics, response to therapy, and outcomes. Cancer.

[36] Richard-Carpentier G, Rausch CR, Sasaki K, et al. Characteristics and clinical outcomes of patients with acute myeloid leukemia with inv(3)(q21q26.2) or t(3;3)(q21;q26.2). *Haematologica.* 2023. 10.3324/haematol.2022.282030 PMC10483357 36951163

[37] Saliba AN, Kaufmann SH, Stein EM, et al. Pevonedistat with azacitidine in older patients with TP53-mutated AML: a phase 2 study with laboratory correlates. *Blood Adv.* 2022. 10.1182/bloodadvances.2022008625 PMC10230164 36315007

[38] Ye H, Adane B, Khan N (2016). Leukemic stem cells evade chemotherapy by metabolic adaptation to an adipose tissue niche. Cell Stem Cell.

[39] Stevens BM, Jones CL, Pollyea DA (2020). Fatty acid metabolism underlies venetoclax resistance in acute myeloid leukemia stem cells. Nat Cancer.

