# Supplementary material for: A phase 1/2 study of azacitidine, venetoclax and pevonedistat in newly diagnosed secondary AML and in MDS or CMML after failure of hypomethylating agents
Source: J Hematol Oncol. 2023 Jul 8;16:73. doi: 10.1186/s13045-023-01476-8 (PMC10329789; doi:10.1186/s13045-023-01476-8)

**Title:** A Phase 1/2 Study of Azacitidine, Venetoclax and Pevonedistat in Newly Diagnosed Secondary AML and in MDS or CMML after Failure of Hypomethylating Agents

**Running title:** Azacitidine, venetoclax and pevonedistat in AML, MDS and CMML

Supplementary Information

**Supplemental Methods**

**CyTOF analysis**

**Samples**

Peripheral blood (PB) and bone marrow (BM) samples were obtained from patients enrolled in a phase 1/2 clinical trial investigating the safety and efficacy of azacitidine, venetoclax and pevonedistat combination therapy. PB and BM samples were processed as previously described.^1^

**Sample barcoding**

The PB and BM samples were barcoded using the Cell-ID 20-Plex Pd barcoding kit (Standard Bio Tools, San Francisco, CA), which utilizes triple combinations of six different palladium isotopes. In brief, the cells were fixed, washed once in 1X barcode perm buffer, and resuspended in 800 μL barcode perm buffer. Barcodes were thawed at room temperature, quick-spun, and resuspended in 100 μL barcode perm buffer. The barcodes were transferred to the appropriate samples, mixed thoroughly, and incubated at room temperature for 30 minutes. The Samples were then washed three times in cell staining buffer (0.5% bovine serum albumin (BSA) in PBS. The cells then were resuspended in 100 μL PBS, transferred, and collected in a single tube. Before staining, the cells were counted, spun down, and resuspended in a cell staining buffer.

**CyTOF staining and data acquisition:**

Prior to staining, cells were labeled with 5-Iodo-2’-deoxyuridine (IdU) (Acros Biosystems) to mark cells in S-phase of the cell cycle. Cells were incubated at a final concentration of 10 μM IdU for 30 minutes at 37°C/5% CO2 and then washed twice in CSB. The pooled samples were incubated with Fc blocker for 10 minutes using Human TruStain FcX™ solution (Biolegend). Then, a freshly prepared antibody mixture against cell surface markers was added to cell mixture, and the samples were incubated for 30 minutes at RT. The antibodies and corresponding metal tags used in the 51-parameter CyTOF panel are shown in the table below.

**
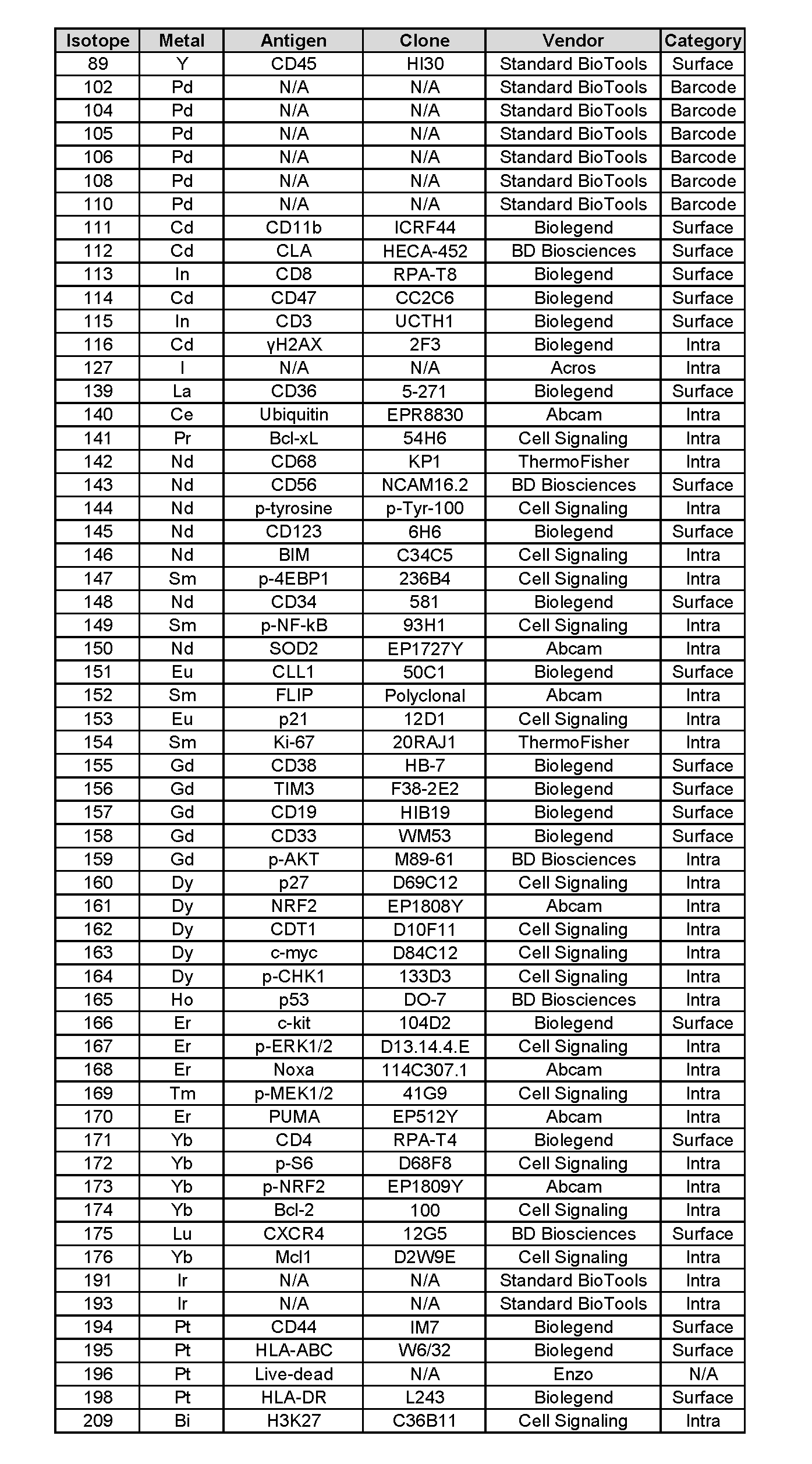
**After incubation the cells were washed twice with cell staining buffer, fixed in 1.6% PFA for 10 minutes at room temperature, and permeabilized in 90% methanol at -20°C for 1 hour. Cells were then washed twice in cell staining buffer and stained with intracellular (IC) antibodies for 30 minutes at 4°C, resuspended in intercalator solution (Standard Bio Tools, San Francisco, CA), and incubated overnight at 4°C. Finally, the cells were washed twice with CSB and once with double-distilled water, and data was acquired on a Helios mass cytometer.

**Data analysis**

The pooled sample sets were deconvoluted using the Premessa R-package or Debarcoder software (Standard Bio Tools, San Francisco, CA). The deconvoluted samples were cleaned up using Flowjo version 10.8.1, and single live cells were selected and exported by excluding calibration beads, gating on singlets based on DNA content and event length, and excluding dead cells by selecting cells having low cisplatin uptake. High-dimensional analysis was performed using the Omiq.ai platform and uniform manifold approximation and projection (UMAP) was used for dimension reduction. Differential expression analysis was performed using Seurat package^2^ and 100 cells from each sample were randomly downsampled violin plots were generated using R using ggplot2^3^. Normality of data was tested before analysis. If the dataset was found to be normally distributed, we used t-test for parametric analysis. Otherwise, for nonparametric analysis, we used Mann-Whitney U test.

**References**

1. Muftuoglu M, Li L, Liang S, et al. Extended live-cell barcoding approach for multiplexed mass cytometry. *Sci Rep*. Jun 11 2021;11(1):12388. doi:10.1038/s41598-021-91816-w

2. Satija R, Farrell JA, Gennert D, Schier AF, Regev A. Spatial reconstruction of single-cell gene expression data. *Nature biotechnology*. 2015;33(5):495-502.

3. Wickham H. ggplot2. *Wiley interdisciplinary reviews: computational statistics*. 2011;3(2):180-185.

**Supplemental Figure 1.** Overall survival of the AML cohort, stratified by cytogenetic risk.

**Supplemental Figure 2.** Overall survival of the MDS/CMML cohort.

**Supplemental Figure 3.** Comparison of marker expression levels in AML blasts and monocytic cells between pretreatment and day 2 of therapy using CyTOF analysis

**Supplemental Figure 4.** Comparison of marker expression levels in AML blasts and monocytic cells between pretreatment and day 21 of therapy using CyTOF analysis

**Supplemental Figure 1.** Overall survival of the AML cohort, stratified by cytogenetic risk.

**Supplemental Figure 2.** Overall survival of the MDS/CMML cohort.

**Supplemental Figure 3.** Comparison of marker expression levels in AML blasts and monocytic cells between pretreatment and day 2 of therapy using CyTOF analysis. **A.)** Live, singlet cells from pretreatment, day 2 and day 21 samples were subjected to UMAP dimension reduction and projected in two dimensions. AML blasts and monocytes were identified based on their expression of specific markers and are shown in purple and blue, respectively. **B.**) Violin plots depicting the expression levels of NOXA and CD36 in AML blasts (left panel) and NOXA and pNRF2 in monocytic cells (right panel). Approximately 100 cells each from pre- and post-treatment sample were randomly down-sampled for comparative analysis and plotted using violin plots to visualize the distribution of marker expression levels. Only proteins with significant change in expression are shown.


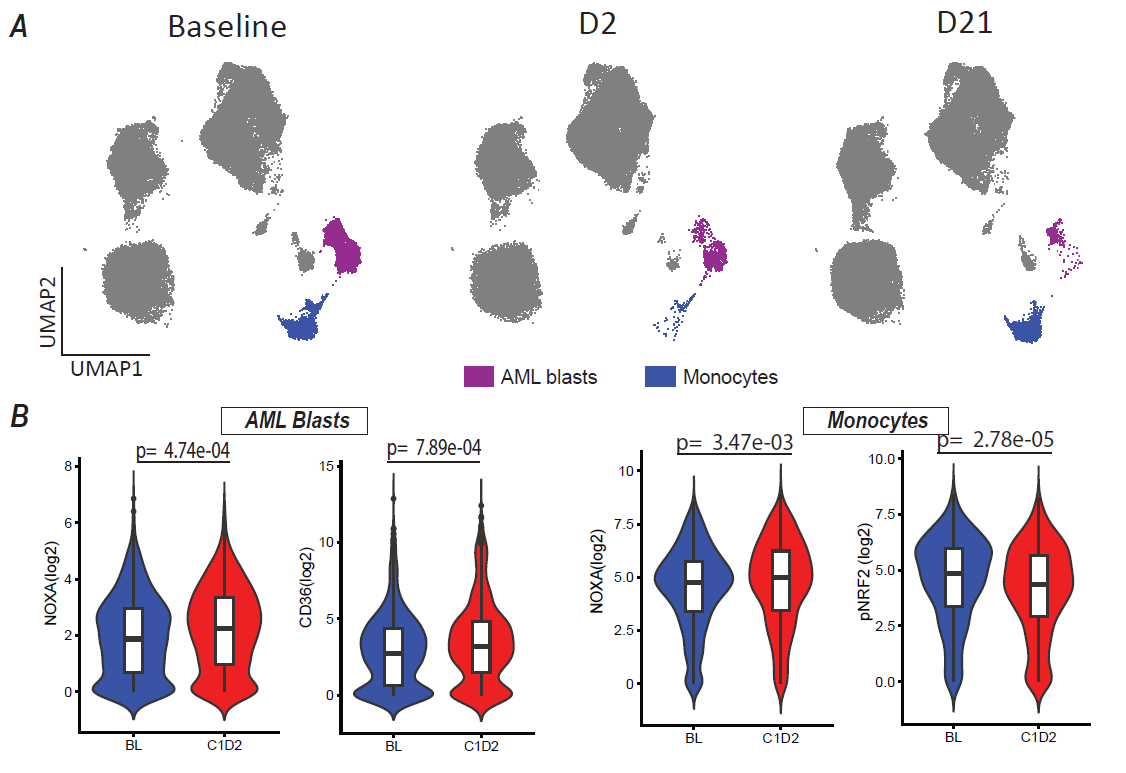


**Supplemental Figure 4.** Comparison of marker expression levels in AML blasts and monocytic cells between pretreatment and day 21 of therapy using CyTOF analysis. **A)** Violin plots showing the expression levels of MCL1, FLIP, CD36 and c-Myc in AML blasts. **B.)** Violin plots showing the expression levels of MCL1 and pNRF2 in monocytic cells. To compare marker expression levels, approximately 100 cells were randomly down-sampled from both pre- and post-treatment samples, and their distribution was visualized using violin plots. Only proteins with significant change in expression are shown.


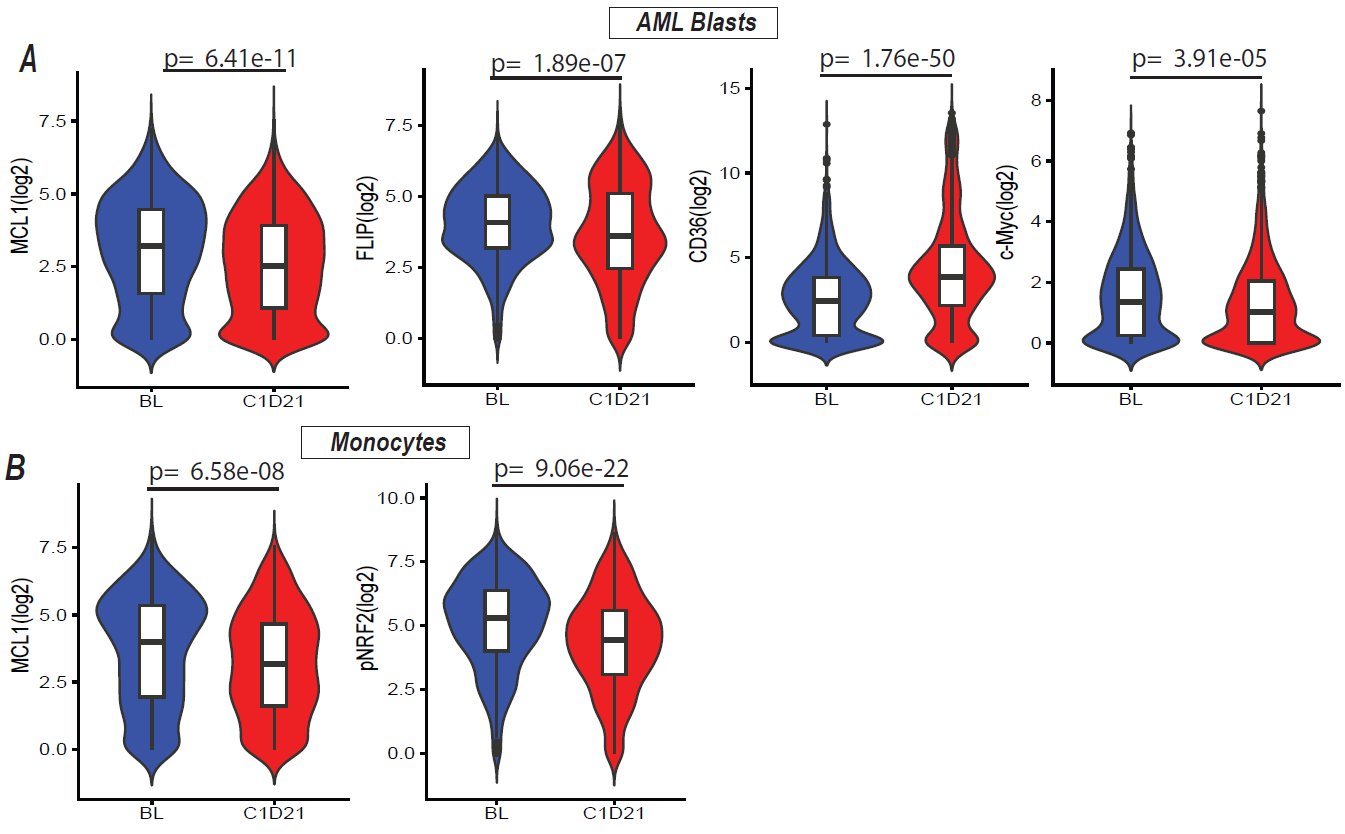

Supplement: Supplementary file 1 — Additional file 1. Supplemental methods and figures. [file 13045_2023_1476_MOESM1_ESM.docx]
